# Fabrication of capacitive pressure sensor using single crystal diamond cantilever beam

**DOI:** 10.1038/s41598-019-40582-x

**Published:** 2019-03-18

**Authors:** Jiao Fu, Tianfei Zhu, Yan Liang, Zhangcheng Liu, Ruozheng Wang, Xiaofan Zhang, Hong-Xing Wang

**Affiliations:** Institute of Wide Band Gap Semiconductors, Xi’an Jiaotong University, Xi’an, China

## Abstract

Fabrication of single crystal diamond capacitive pressure sensor is presented. Firstly, the single crystal diamond cantilever beam was formed on HPHT diamond substrate by using selective high-energy ion implantation, metal patterning, ICP etching and electrochemical etching techniques. Secondly, on this diamond cantilever beam, the desired electrode patterns were processed with photolithography and metal evaporation methods. Furthermore, the displacements of cantilever beam under different pressure conditions were investigated by atomic force microscopy. The capacitance-voltage curves of single crystal diamond cantilever beam and substrate under different force loading conditions were measured by using Agilent B1505A parameter analyzer. The results show that sensitivity increases with the enlargement of electrode area of cantilever beam, and decreases with the rise of measurement frequency.

## Introduction

Pressure sensors are realized by a variety of function types including piezoelectricity, piezoresistivity, capacitance, bonded strain gauges and others^1^. Among them, capacitive pressure sensors obtain much attention due to its higher measurement sensitivity, decreased temperature sensitivity, reduced power consumption and better stability^2^. These advantages make it have greater potential for commercial applications. Conventionally, silicon based pressure sensors were widely used for normal operational environments. However, there are increasing demands of sensor to be used in harsh environments with high temperatures, high oxidizing, high radiations and strong corrosion. In such conditions, silicon is not suitable^3^. Thus, it is mandatory to search for an appropriate sensor material capable to overcome the above mentioned problem.

Diamond is a preferred material for such applications due to its wide band gap energy, high electric breakdown field, high carrier mobility and low dielectric constant^4–7^. Especially, it has excellent physical and chemical properties, including high mechanical hardness, Young’s modulus, corrosion resistance and low friction coefficient^8^. In addition, a small neutron cross section of diamond allows it to experience low degradation in radioactive environments. Moreover, diamond has highest known thermal conductivity at room temperature and exhibits a good thermal conductivity over a wide temperature range. Thus, diamond is suitable candidate material for pressure sensors which can be applied to extreme environment such as the combustion chamber of rocket engine and gas-fired boilers for pressure monitoring. The reported diamond sensors are mainly fabricated on polycrystalline or nanocrystalline films due to the commercially available SCD is much smaller and it is difficult to grow SCD on the hetero-substrates. Compared to SCD, polycrystalline or nanocrystalline diamond suffer from degradation in performance, poor reproducibility and difficulties in electrical conductivity control because of the existence of grain boundaries, impurities and large stress in the films. Thus, SCD is the promising characteristics for sensor devices. In recent years, H.Yamada *et al*. fabricated 2-inch size SCD mosaic wafer by using a cloning technique^9^, and Matthias Schreck *et al*. reported that SCD plate with a diameter of 92 mm was synthesized by heteroepitaxy on Ir/YSZ/Si (001) substrate^10^. There are also several companies producing inch size SCD diamond substrates in the world. All these large SCD form the base for developing devices. Nevertheless, diamond is difficult to be micromachined due to its mechanical and chemical stability.

In this paper, a fabrication of micro-SCD capacitive pressure sensor by using selective high-energy ion implantation, ICP etching and electrochemical etching, metal evaporation techniques was successfully carried out.

## Experiment

Fabrication of single-crystal diamond cantilever beam began from HPHT Ib (001) SCD substrate, which was selectively implanted by carbon ions with energy of 3 MeV^11^. Then a homoepitaxial layer was grown on the substrate by a microwave plasma chemical vapor deposition system at 1175 °C. During growth, the damaged layer caused by ion implantation was transformed into graphite, providing a sacrificial layer for the formation of cantilever beam structures for subsequent processing^12,13^. Then, aluminum film with a thickness of 1 μm was formed on the substrate by conventional photolithography and magnetron sputtering techniques to define the beam structures as a mask for inductively coupled plasma etching. After that, the sacrificial layer was removed to release the cantilever beam by non-contact electrochemical etching system according to the procedure in ref.^14^. Finally, the diamond cantilever beam was treated with photolithography and metal evaporation techniques to pattern the desired electrode patterns for electrical measurements of capacitive pressure sensor. The displacement variations of cantilever beam versus force loading and capacitance-voltage curves under different force loading conditions were measured in air at room temperature by using atomic force microscopy and Agilent B1505A parameter analyzer, respectively.

## Result and Discussion

**Figure 1:**
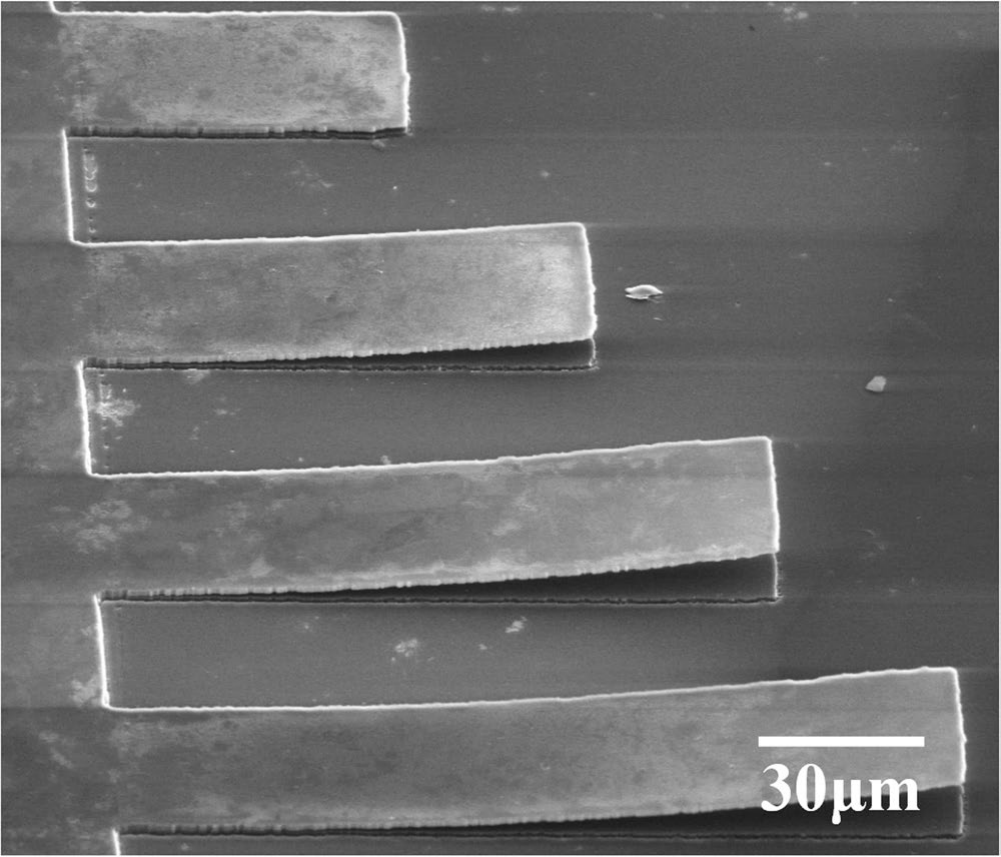
SEM image of free standing SCD cantilever beam.

After ion implantation, an accepted quality diamond epitaxial layer can be grown on the damaged surface layer^6^. The sacrificial layer induced by ion implantation was removed by electrochemical etching successfully. Figure 1 shows an array of free standing SCD cantilever beam SEM image with width of 30 μm and lengths of 65 μm, 95 μm, 125 μm, 155 μm, reflectively, which were all supported on the diamond substrate, revealing that SCD cantilever beam was obtained through ion implantation, ICP and electrochemical etching techniques. The air gap between the diamond cantilever beam and substrate can be clearly observed, ensuring the vertical motional function possible. In addition, the dimensions of cantilever beam can be controlled well using the above process.

**Figure 2:**
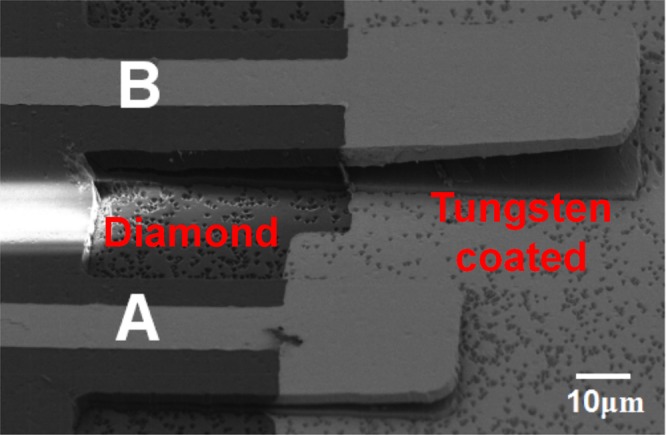
SEM image of cantilever beam patterned with tungsten electrode.

In order to realize capacitive pressure sensor, the sample was patterned with tungsten film. Figure 2 clearly exhibits a cantilever beam structure with electrode. The dark grey area indicates no tungsten coating area and the white area shows tungsten coating area as an electrode. Both the cantilever beams and the substrate surface underneath the cantilever beam are coated with tungsten during evaporation. The two cantilever beams are labeled with A and B. The dimensions of cantilever A is 65 × 30 × 2.5 µm^3^ with electrode area of 35 × 30 µm^2^ and that of cantilever B is 95 × 30 × 2.5 µm^3^ with electrode area of 50 × 30 µm^2^.

**Figure 3:**
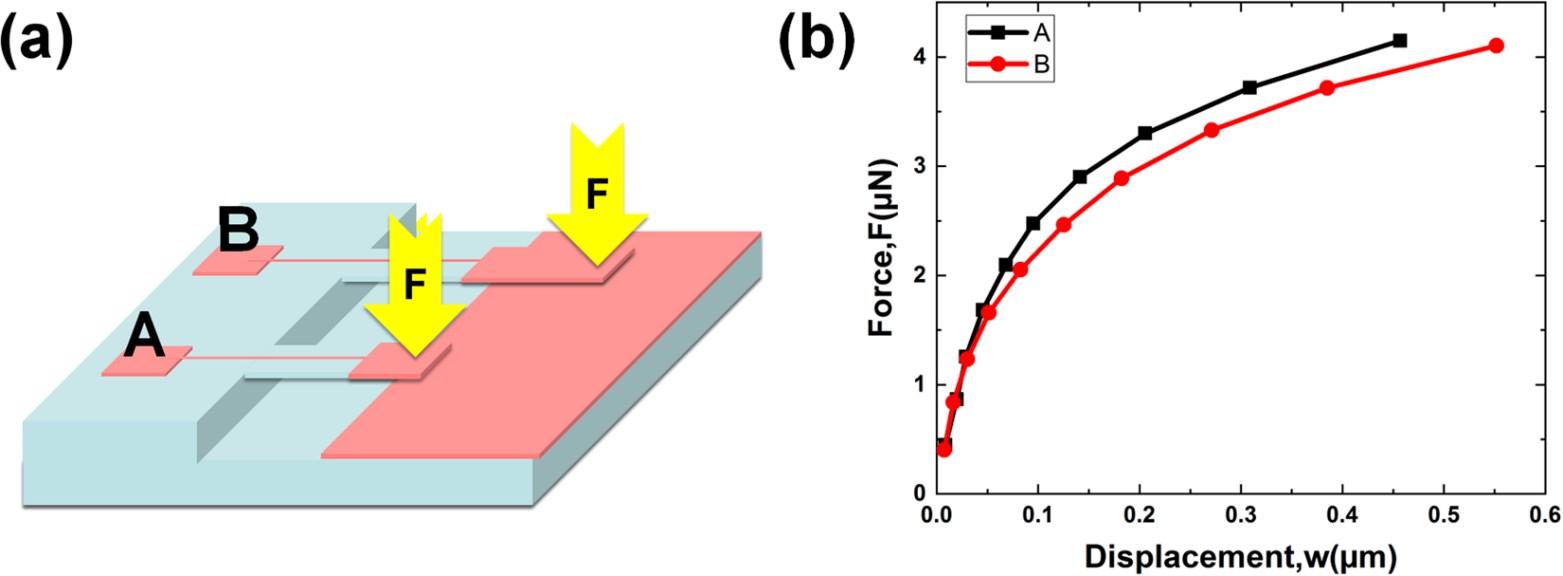
(**a**) Schematic of AFM bending test of SCD cantilever beam. (**b**) Force–displacement characteristics of cantilever beam A and B from bending test.

A small force loading was applied at the tip of SCD cantilever beam to characterize the displacement variation as a function of force loading by AFM system. Figure 3(a) shows the schematic of AFM bending test. Figure 3(b) shows the F-w curve under different force loading conditions of cantilever beam A and B, displaying that the SCD cantilever beam did not show any fracture during the measurement.

The F-w curve function was fitted as:

$$\begin{array}{rcl}{{\rm{F}}}_{{\rm{A}}} & = & 0.001\,{\rm{lnw}}+0.0116\\ {{\rm{F}}}_{{\rm{B}}} & = & 0.0009\,{\rm{lnw}}+0.0105\end{array}$$

where, F stands for the force loading applied by AFM, w stands for displacement of cantilever beam.

A variation of capacitor is realized with the flexible plate and a fixed plate. The distance between plates equals *d* and the original plates distance without force loading becomes *d* + *w*. The capacitive behavior of the plate was studied under different plates distances. The capacitance is calculated using the following formula:

$$C=\frac{\varepsilon S}{d}$$

where, ε stands for permittivity, and S is the electrode area with tungsten coated.

**Figure 4:**
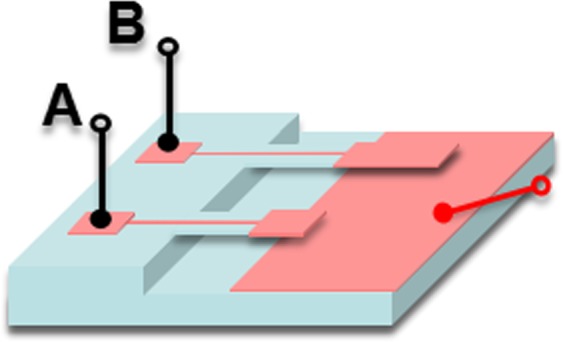
Schematic of capacitance test by using Agilent B1505A parameter analyzer.

**Figure 5:**
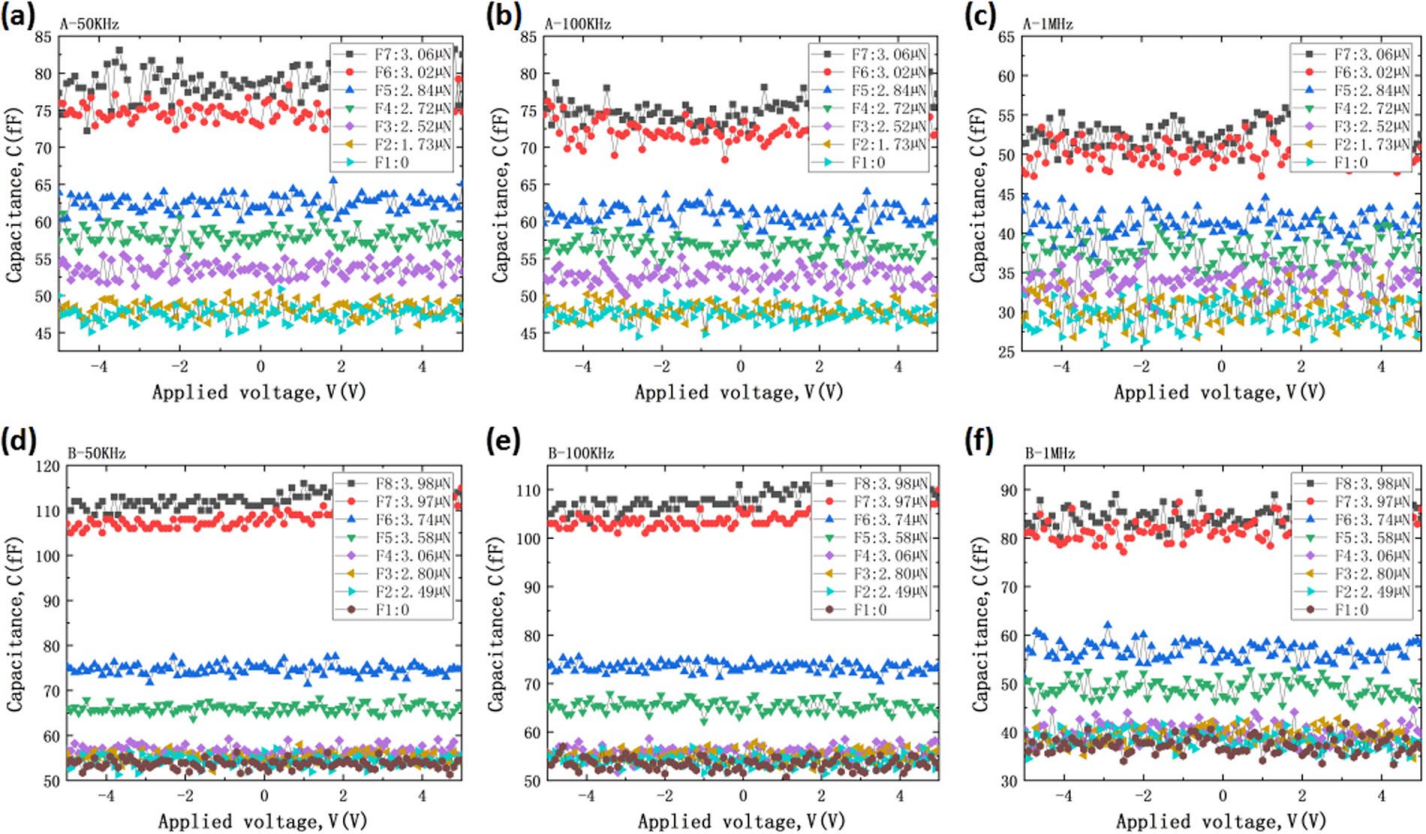
Capacitance-voltage characteristics under different plates distance conditions of cantilever beam A at frequency of (**a**) 50 KHz, (**b**) 100 KHz, (**c**) 1 MHz and cantilever beam B at frequency of (**d**) 50 KHz, (**e**) 100 KHz, (**f**) 1 MHz.

Figure 4 displays the schematic of capacitance test. The black and red lines act as two different electrodes. In this case, the plates distance was controlled by micro-needle. Based on the F-w data in Fig. 3, the C-V curves of cantilever beam A and B under different force loading conditions were measured and presented in Fig. 5, during which the voltage frequencies were 50 KHz, 100 KHz and 1 MHz, respectively. Figure 5 clearly shows that the capacitance value is almost a constant at same force loading condition, and its average value increases steady as force loading increases. The capacitance value fluctuation of cantilever beam B is smaller than that of A.

**Figure 6:**
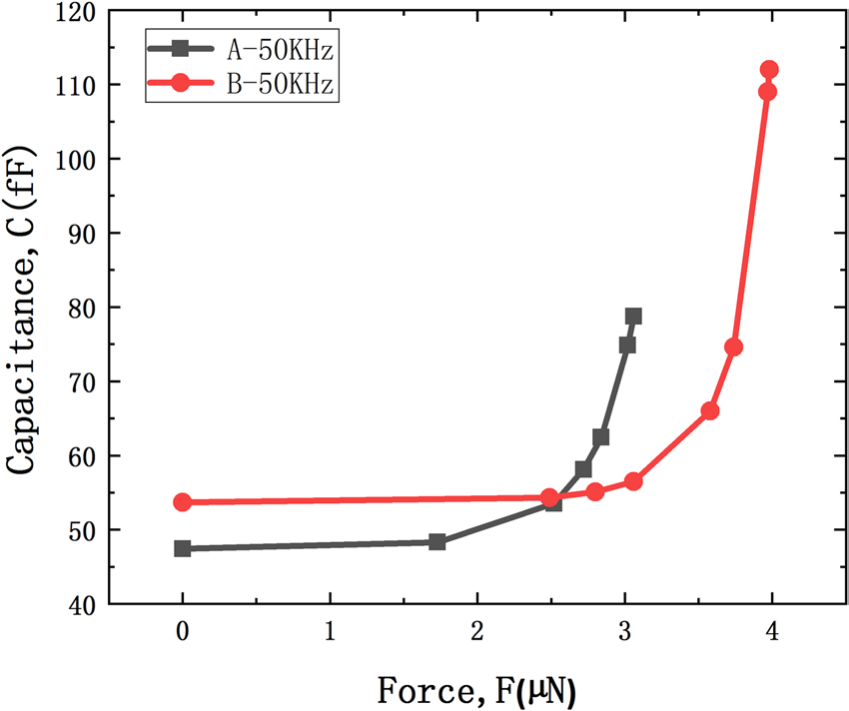
Cantilever beam capacitance as a function of force loading measurement at A and B.

Figure 6 shows the capacitance value of the cantilever beam A and B as a function of force loading measurement at frequency of 50 KHz. Capacitance value was increase with increasing applied force loading. At the beginning, there is a very small change in the capacitance due to the cantilever beam was bent in the elastic region under small force loading condition and the high Young’s module of diamond. Then the capacitance was increased dramatically because the distance between plates decreased remarkably in non-elastic deformation region and capacitance is proportional to 1/d.

**Figure 7:**
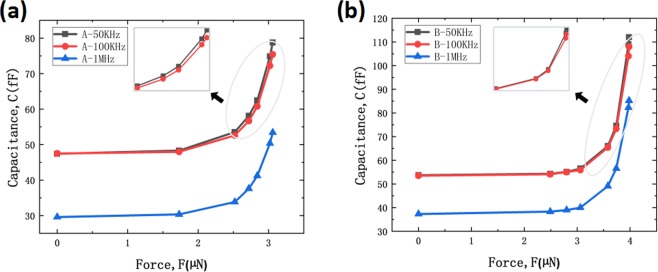
Capacitance versus force loading measurement at frequency of 50 KHz, 100 KHz, 1 MHz of (**a**) cantilever beam A and (**b**) cantilever beam B.

After testing the capacitive behavior at frequency of 50 KHz, the same process was conducted at frequencies of 100 KHz, 1 MHz for cantilever beam A and B, respectively, as shown in Fig. 7. Capacitance was increased slowly at the beginning then increased dramatically when force loading increases as explained before. Capacitance value was found to be larger under low frequency of 50 kHz, indicating that our capacitive pressure sensor is more suitable for working under low frequency. The tendency of capacitance variation was not affected by frequency under the same force loading condition.

**Figure 8:**
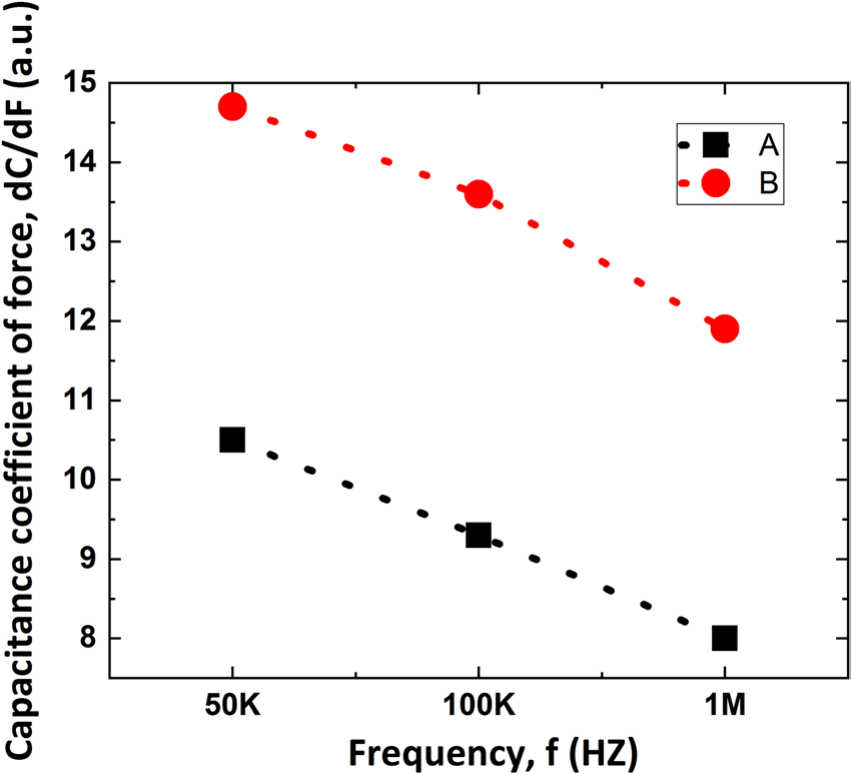
Sensitivity curve of cantilever beam A and B with frequency of 50 KHz, 100 KHz, 1 MHz.

Figure 8 shows the plots of sensitivity results of cantilever beam A and B with frequency of 50 KHz, 100 KHz, 1 MHz. It is clear that the sensitivity increases with the enlargement of electrode area of cantilever beam, and decreases with the rise of measurement frequency.

## Conclusion

Micro-SCD capacitive pressure sensor on HPHT diamond substrate has been successfully fabricated and investigated. The C-F curve of SCD cantilever beam shows that the capacitance increases when force loading increases. Sensitivity is proportional to electrode area of cantilever beam and is inversely proportional to measurement frequency. Capacitive pressure sensor fabricated by diamond can be applied in harsh environment especially at high temperatures, high oxidizing, high radiation and corrosive environments.
